# Isolation, Functional Characterization and Transmissibility of p3PS10, a Multidrug Resistance Plasmid of the Fish Pathogen *Piscirickettsia salmonis*

**DOI:** 10.3389/fmicb.2018.00923

**Published:** 2018-05-08

**Authors:** José Saavedra, Maritza Grandón, Juan Villalobos-González, Harry Bohle, Patricio Bustos, Marcos Mancilla

**Affiliations:** ^1^Laboratorio de Diagnóstico y Biotecnología, ADL Diagnostic Chile SpA, Puerto Montt, Chile; ^2^Escuela de Bioquímica, Facultad de Ciencias, Universidad Austral de Chile, Valdivia, Chile

**Keywords:** *Piscirickettsia salmonis*, piscirickettsiosis, antibiotic resistance, tetracycline, plasmid

## Abstract

Antibiotic resistance is a major public health concern due to its association with the loss of efficacy of antimicrobial therapies. Horizontal transfer events may play a significant role in the dissemination of resistant bacterial phenotypes, being mobilizable plasmids a well-known mechanism. In this study, we aimed to gain insights into the genetics underlying the development of antibiotic resistance by *Piscirickettsia salmonis* isolates, a bacterial fish pathogen and causative agent of salmonid piscirickettsiosis, and the main target of antibiotics used in Chilean salmon farming. We provide experimental evidence that the plasmid p3PS10, which harbors multidrug resistance genes for chloramphenicol (*cat2*), tetracyclines [*tet*(31)], aminoglycosides (*sat1* and *aadA1*), and sulfonamides (*sul2*), is carried by a group of *P. salmonis* isolates exhibiting a markedly reduced susceptibility to oxytetracycline *in vitro* (128–256 μg/mL of minimal inhibitory concentration, MIC). Antibiotic susceptibility analysis extended to those antibiotics showed that MIC of chloramphenicol, streptomycin, and sulfamethoxazole/trimethoprim were high, but the MIC of florfenicol remained at the wild-type level. By means of molecular cloning, we demonstrate that those genes encoding putative resistance markers are indeed functional. Interestingly, mating assays clearly show that p3PS10 is able to be transferred into and replicate in different hosts, thereby conferring phenotypes similar to those found in the original host. According to epidemiological data, this strain is distributed across aquaculture settings in southern Chile and is likely to be responsible for oxytetracycline treatment failures. This work demonstrates that *P. salmonis* is more versatile than it was thought, capable of horizontally transferring DNA, and probably playing a role as a vector of resistance traits among the seawater bacterial population. However, the low transmission frequency of p3PS10 suggests a negligible chance of resistance markers being spread to human pathogens.

## Introduction

Mammal husbandry and aquaculture are human activities that demand antimicrobials at a large scale, not only as a veterinary tool for disease management, but also to meet animal welfare directives. The environment surrounding these production settings, especially sediments, contains residues of antibiotics, yielding favorable conditions for the selection and emergence of resistant types. In this regard, it has been suggested that the environment plays an important role as a reservoir of resistant bacteria and genes that might transform susceptible cells into resistant pathogenic bacteria (Berendonk et al., 2015). The salmon industry in Chile is threatened by this situation. Several studies have accounted for the presence of environmental non-wild-type bacterial strains and resistance markers near Chilean marine aquacultures (Miranda and Zemelman, 2002; Miranda et al., 2003; Miranda and Rojas, 2007; Buschmann et al., 2012; Shah et al., 2014). Therefore, it has been hypothesized that aquaculture settings may serve as a source of antibiotic resistance genes (ARG), thereby enhancing the likelihood of growing resistance to antibiotics in human and animal pathogens (Cabello et al., 2016).

Piscirickettsiosis, also named salmonid rickettsial septicemia or SRS, is a severe bacterial disease affecting reared salmonids worldwide. The incidence of the disease is particularly high in southern Chile, where it is triggered by notably virulent strains of *Piscirickettsia salmonis*. In this country, economic losses attributed to SRS have been estimated at more than half a billion dollars annually (Maisey et al., 2016). *P. salmonis* is a Gram-negative, facultative intracellular pathogen that has shown remarkable resilience to be controlled by means of conventional strategies (Rozas and Enríquez, 2014). Attempts to limit outbreaks and pathogen dissemination with antimicrobials have probably led to the emergence of isolates with reduced susceptibility to different therapeutic agents (Henríquez et al., 2015). This issue represents an important concern since only a few active principles are authorized for SRS treatments, with florfenicol (FFC) and oxytetracycline (OTC) being the main drugs used (SERNAPESCA, 2016).

Thus far, it is known that the *P. salmonis* genome encompasses a chromosome of about 3.2 Mb and at least three plasmids of medium size (Pulgar et al., 2015). Comparative studies have shown that *P. salmonis* essential genes are encoded by the chromosome, including those necessary for virulence (Bravo and Martinez, 2016). However, accessory genes contained in additional replicons such as plasmids carried by *P. salmonis* field isolates have not yet been studied in detail. As recently reported, our laboratory accomplished the sequencing of *P. salmonis* AY3800B (Bohle et al., 2017). This isolate is representative of a genovariant that displays a phenotype associated with high *in vitro* minimal inhibitory concentrations (MIC) of OTC (128–256 μg/mL). Such high *in vitro* OTC MIC levels will probably lead to treatment failures as these greatly exceed the maximum plasma levels achieved when salmonids are medicated with OTC (Elema et al., 1996). During sequence analysis, a plasmid designated as p3PS10 showed to be unique to AY3800B. Interestingly, this plasmid encodes several antibiotic resistance markers, along with a cluster of type 4 secretion system (T4SS) genes probably related to the ability of self-transference. In this study, we address the functional characterization of the first episome isolated from *P. salmonis* and discuss the implications of its potential transmissibility to other bacteria.

## Materials and methods

### Bacterial strains, growth conditions and nucleic acid purification

Bacterial strains and plasmids used in this study are listed in Table 1. *Escherichia coli* strains were grown on standard trypticase soy broth (TSB, BD) at 37°C. For *P. salmonis* growth, plates filled with nutrient-rich medium ADL-PSA were left for 5 days at 18°C (Henríquez et al., 2015). Bacteria were subsequently used for genomic DNA purification with the GeneJet Genomic DNA Purification Kit according to the manufacturer's instructions (Thermo Fisher Scientific). DNA concentration and quality were assessed by means of a fluorometric method (Qubit, Thermo Fisher Scientific) and agarose gel electrophoresis, respectively. Plasmid miniprep purifications were carried out using a commercial kit (Quiaprep, Qiagen). To collect enough material for restriction digestion analysis, a midiprep was subsequently done (Heilig et al., 2001). Plasmids precipitated with isopropanol and pelleted by centrifugation were finally purified with a commercial kit (GeneJet). Internal procedure PGSe-03 “Management of biological and toxic waste,” which is in compliance with the Chilean directive D.S. 148 “Sanitary Regulation for Hazardous Wastes Management,” was followed for the management of biological residues.

**Table 1:** Bacterial strains and plasmids used in the study.

**Name/species**	**Description**	**Reference/source**
pCR2.1TOPO	Cloning plasmid, 3.9 kb in length. Ampicillin and kanamycin resistances.	Invitrogen
pCFC	pCR2.1TOPO derivative containing a 919 bp PCR fragment including *cat2*, amplified with primers CFC_F and R.	This study
pOTC	pCR2.1TOPO derivative containing a 2,611 bp PCR fragment including *tetA*(31)*-tetR* tandem, amplified with primers tet_F and R.	This study
pSTR	pCR2.1TOPO derivative containing a 1,620 bp PCR fragment including *sat1-aadA1*, amplified with primers STR_F and R.	This study
pSUL2	pCR2.1TOPO derivative containing a 1,001 bp PCR fragment including *sul2*, amplified with primers Sul2_F and R.	This study
p3PS10	*P. salmonis* multidrug resistant, low copy number plasmid, 45.7 kb in length.	Bohle et al., 2017
AY3800B	*P. salmonis* isolate carrying the p3PS10 plasmid. LF-89 genogroup.	Bohle et al., 2017
AY3864B	*P. salmonis* isolate recovered from *S. salar* in 2013. LF-89 genogroup.	This study
AY6297B	*P. salmonis* isolate recovered from *S. salar* in 2015. LF-89 genogroup.	This study
AY6532B	*P. salmonis* isolate recovered from *S. salar* in 2015. LF-89 genogroup.	This study
LF-89	*P. salmonis* wild-type reference strain. Recovered from Coho salmon in 1989.	Fryer et al., 1992. American Type Culture Collection (ATCC).
PM32597B1	*P. salmonis* field isolate, recovered from Coho salmon in 2012. LF-89 genogroup.	Bohle et al., 2014
PM63907	*P. salmonis* field isolate recovered from *S. salar* in 2015. LF-89 genogroup.	This study
PM15972A1	*P. salmonis* wild-type field isolate, EM-90 genogroup.	Bohle et al., 2014
PM15972A1 p3PS10	*P. salmonis* transconjugant resulting from a mating assay between AY3800B and PM15972A1 isolates.	This study
PM32597B1 p3PS10	*P. salmonis* transconjugant resulting from a mating assay between AY3800B and PM32597B1 isolates.	This study
PM63907 p3PS10	*P. salmonis* transconjugant resulting from a mating assay between AY3800B and PM63907 isolates.	This study
*E. coli* TOP10	Cloning strain. Susceptible to OTC and CFC, reduced susceptibility to STR.	Invitrogen
*E. coli* DH5α	Cloning strain. Susceptible to STR.	Invitrogen
*E. coli* ATCC 25922	ATCC reference strain for antibiotic susceptibility studies.	ATCC
*E. coli* p3PS10	TOP10 derivative, multidrug resistant strain containing the plasmid p3PS10.	This study
*E. coli* pCFC	TOP10 derivative containing the plasmid pCFC.	This study
*E. coli* pOTC	TOP10 derivative containing the plasmid pOTC.	This study
*E. coli* pSTR	DH5α derivative containing the plasmid pSTR.	This study
*E. coli* pSUL2	TOP10 derivative containing the plasmid pSUL2.	This study

### PCR amplification, molecular cloning and restriction analysis

Cloning of putative resistance markers was done using the PCR2.1 TOPO TA Cloning Kit (Invitrogen). PCR fragments comprising putative native promoter and coding sequences (CDS) were amplified with primers listed in Table 2. The PCR mix contained 2.5 μL of buffer, 1.0 μL of 50 mM MgCl_2_, 0.2 μM of each primer and 1 U of Taq Platinum in a final volume of 25 μL. PCR conditions were as follows: initial denaturation at 95°C for 5 min, followed by 30 cycles of denaturation at 95°C for 15 s, annealing at 60°C for 15 s, extension at 72°C for 15 s, with a final extension at 72°C for 5 min. Primers p3PS10_F and R and conditions similar to these described above were used to verify the presence of p3PS10 in selected isolates of our *P. salmonis* strain collection. For restriction analysis, 0.5 μg of plasmid DNA were digested with *Eco*RI or *Bam*HI for 1 h at 37°C. Samples were separated by 1% agarose gel electrophoresis at 110 V for 30 min.

**Table 2:** Oligonucleotides and probes used in the study.

**Name**	**Sequence (5′-3′)**
rpoD_U	GCGCTCAGTACGTTGTAACGCGTGACACCATCAAA GCGAAAGGTCGCAGTCACGCTACCGCTCAGGAAGA GATCCTGAAACTGTCTGAAG
rpoD_P	FAM/ATCAAAGCGAAAGGTCGCAGTCAC/IABkFQ
rpoD_F	GCGCTCAGTACGTTGTAA
rpoD_R	CTTCAGACAGTTTCAGGATCTC
BC_U	GCAGCCTCTATTCAACTTGAA AGTGGTTGGAATAGCATTAAAAATATTTTC GCGGTGAGTGATGAAGCAGCGGATGT CAGTATTTCAGACTTTGTTCG
BC_P	FAM/TCCGCTGCTTCATCACTCACCG/IABkFQ
BC_F	GCAGCCTCTATTCAACTTG
BC_R	CGAACAAAGTCTGAAATACTGA
tetR_U	GAGAAAATGCGAAAAGCTTTCGTAG GGCGTTATTAAGTCACCGAGATGCGGCA AAAATCCATTTAGGAACAAGACCC
TetR_P	HEX/CGCATCTCGGTGACTTAATAACGCC/IABkFQ
TetR_F	GGGTCTTGTTCCTAAATGG
TetR_R	GCGAGAAAATGCGAAAAG
p3PS10_F	AATCAAGACCGGTTTTCTGC
p3PS10_R	CGAAAGCTTTTCGCATTTTC
tet_F	AGACGGTATTCGTGGCAAAG
tet_R	TTTGCAATGTGCAATTTGGT
CFC_F	TACGGGAGGAATAGCTGGAA
CFC_R	CACCATTCCTTCCGGATACA
STR_F	TCAACAGATCGCGCATAGTC
STR_R	CATCGTGCAAGCAGGATAGA
Sul2_F	GGCTCTTGCAAGTTTTGGAT
Sul2_R	CCGAATGTGCAGTTAACGAAT
	

### Plasmid copy number determination

qPCR-based parameters were applied for the calculation of p3PS10 plasmid copy numbers (PCN; Skulj et al., 2008). Briefly, aliquots of broth cultures of AY3800B, *E. coli* p3PS10, and *E. coli* pOTC were each collected after 24, 48, 72, 96, and 120 h of cultivation at 18°C for *P. salmonis* or 37°C for *E. coli*. Bacterial disruption was accomplished by heating the samples to 95°C for 10 min in PCR water. qPCR were done using the primer pairs tetR_F and R, BC_F and R, and the corresponding probes tetR_P and BC_P. For *E. coli* strains, primers and probe targeting the *rpoD* gene as a single-copy chromosomal marker were used. PCR conditions were those recommended for TaqMan Gene Expression Master Mix (Applied Biosystems): a holding stage at 50°C for 2 min, enzyme activation at 95°C for 10 min, followed by 40 cycles of 95°C for 15 s and 60°C for 1 min. Calibration curves were obtained using log_10_ dilutions of ultramers tetR_U, rpoD_U and BC_U (Table 2). Efficiencies of PCR reactions were taken into account for PCN calculations derived from plasmid/chromosomal quotients as described by Skulj and colleagues.

### Whole-genome sequencing and annotation

The genomes of OTC-resistant AY3864B, AY6297B, and AY6532B *P. salmonis* LF-89-like isolates were sequenced using Pacific Bioscience (PacBio) chemistry. For AY3864B, Illumina technology (HiSeq 2000 platform, RRID:SCR_010233) was utilized additionally. Libraries were prepared using the kits recommended by the manufacturers: DNA Polymerase Binding Kit P6 v2 for PacBio and Nextera for Illumina. Sequencing was performed at Macrogen Inc., Seoul, South Korea. Long reads were de novo assembled with SMRT Analysis version 2.3.0.1 (PacBio, RRID:SCR_002942) to yield the full sequence of each replicon. Subsequently, read correction was done by mapping Illumina paired-end short reads against the PacBio assembly with CLC Genomic Workbench v6.5 software (CLC bio, RRID:SCR_011853). Annotation was accomplished with the NCBI Prokaryotic Genome Annotation Pipeline using the best-placed reference protein set as the annotation method implemented in GeneMarkS+ revision 3.0 software (RRID:SCR_011930). Sequencing statistics and accession numbers for the sequences deposited in GenBank are found in Supplementary Table 1.

### Sequence analysis

Comparative analyses were carried out with CLC software after the p3PS10 sequence was retrieved from GenBank under the accession number CP013819. Standard algorithms BLASTN and BLASTX at NCBI were also used for sequence analyses. Refinement of antibiotic resistance marker prediction was done with tools to be encountered in the Comprehensive Antibiotic Research Database, CARD^1^ (McArthur et al., 2013). This analysis was extended to the entire genome of AY3800B and the reference LF-89 strains (Pulgar et al., 2015; Bohle et al., 2017). Primer design was done with the Primer3 webtool^2^

### Antibiotic susceptibility profiling

Due to the lack of standardized procedures for the determination of MIC for aquatic microorganisms like *P. salmonis*, we carried out a protocol previously established in our laboratory to this end (Henríquez et al., 2015). This assay relies on the use of PSB medium at 18°C, which supports the growth of *P. salmonis*. This medium is equivalent to the one recommended by the Clinical Laboratory Standards Institute (CLSI) to perform MIC assays for *P. salmonis* (Yañez et al., 2012). Stock solutions of chloramphenicol (CFC), FFC, OTC, streptomycin (STR), and sulfamethoxazole/trimethoprim 19:1 (SXT) (all purchased from Sigma-Aldrich) were prepared following the procedure described in the guidelines (CLSI, 2014a). Solvents for the dilution of antibiotics corresponded to those described in the CLSI document VET03/VET04_S2 (CLSI, 2014b). 96-well microplates were filled with twofold serial dilutions of 0.06–1,024 μg/mL of each antibiotic, then inoculated with approximately 5 × 10^5^ colony forming units/well. MIC were determined with a microplate reader after 96–120 h, considering the lowest concentration of antibiotics that impeded bacterial growth. For the assessment of MIC for *E. coli*, Mueller-Hinton broth with cation-adjusted medium (BD) at 22°C was used along with *E. coli* ATCC 25922 as control strain.

### Mating assays

Donors *P. salmonis* AY3800B and p3PS10-lacking *P. salmonis* (PM15972A1, PM32597B1, PM63907), as well as *E. coli* TOP10 recipient strains were grown to the early exponential phase of their respective growth curves. Aliquots of 1.0 mL (OD_600_ = 1.0) were mixed, centrifuged at 5,000 rpm for 5 min, and resuspended in 0.1 mL of saline before being spread onto a non-selective ADL-PSA plate in the absence of antibiotic. Plates were incubated at 18°C for 24 h. Bacteria were collected by scraping plates and were transferred to 1.0 mL of saline solution, spinned down for resuspension in 0.1 mL of saline and finally spread onto ADL-PSA or TSA plates under selective conditions depending on the recipient. *P. salmonis* transconjugants were recovered after 7 days at 18 or 22°C, and *E. coli* transconjugants after 24 h at 22°C. In the case of *P. salmonis* mating, transconjugants were selected on PSA agar plates plus OTC (50 μg/mL), in some instances after their additional supplementation with oxolinic acid (5 μg/mL). In all experiments performed, donor and recipient cells streaked on plates selective for transconjugants were used as controls. Transconjugant genotypes were confirmed by PCR. Transfer frequencies were measured as the ratio of transconjugants to recipient cells assuming similar numbers of donor and recipient cells.

## Results

### p3PS10 is a multidrug resistance, IncP-related, low-copy-number plasmid

**Figure 1:**
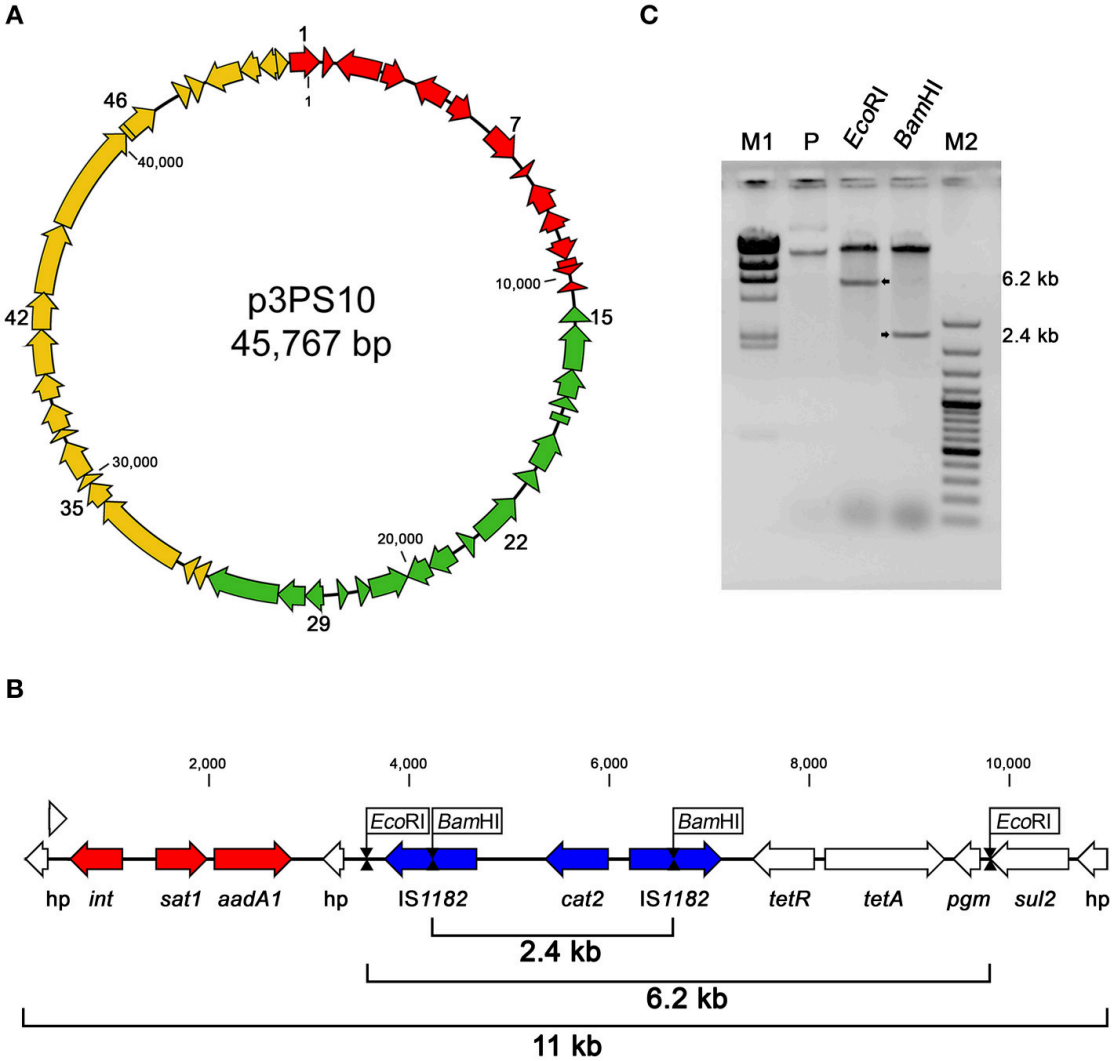
**(A)** Genetic organization of plasmid p3PS10. In red, CDS for putative antibiotic resistance markers. Green arrows represent CDS putatively involved in replication/maintenance. Yellow arrows indicate CDS related to plasmid mobilization. More details on the annotation are found in Table 3. **(B)** Detailed organization of the 11-kb multidrug resistance locus showing integron (red) and transposon (blue) structures. **(C)** Restriction analysis of p3PS10 (inverted colors). Fragments released after *Bam*HI and *Eco*RI digestion are shown in the corresponding lanes (arrows), while the P lane shows the linear and supercoiled forms present in the purified material. M1, Lambda DNA/*Hind*III ladder; M2, GeneRuler 100 bp Plus DNA ladder (both from Thermo Fisher Scientific).

Plasmid p3PS10 is predicted to encode 52 CDS, which are listed in Table 3. According to their hypothetical functions, the entire sequence can be split into three sections: replication/maintenance, transmissibility and antibiotic resistance determinants (Figure 1A). Interestingly, a pair of *tet*(31) tetracycline (TET) resistance genes encoding a transcriptional regulator and an efflux pump protein were found, resembling a canonical array for TET-resistance cassettes (Thaker et al., 2009). In close proximity to the *tet* element we found a gene encoding dihydropteroate synthase (*sul2*), an enzyme associated with reduced susceptibility to sulfonamides, and an acetyltransferase encoded by *cat2*, which likely confers resistance to CFC (Sköld, 2000; Schwarz et al., 2004). Moreover, integron-associated acetyltransferase *sat1*, along with an adenylyltransferase (*aadA1*) related to aminoglycoside resistance were identified (Ramírez et al., 2010). All these CDS were detected within an 11-kb locus (Figure 1B). This region has a complex organization including integron and transposon arrangements. Restriction digestion of p3PS10 with *Eco*RI or *Bam*HI yielded fragments of 6.2 and 2.4 kb, suggesting that the size of the antibiotic resistance locus was accurately estimated from the sequence assembly (Figure 1C). Comparative analysis performed with CARD matched with all the mentioned markers, but also identified five additional chromosomal targets for CFC resistance in sequences of both the wild-type LF-89 and AY3800B strains (Supplementary Table 2).

**Table 3:** Predicted CDS for p3PS10.

**Number**	**CDS**	**Gene**	**Annotation**
1	AVM72_16300	*sul2*	Dihydropteroate synthase
2	AVM72_16305	*pgm*	Phosphoglucomutase (fragment)
3	AVM72_16310	*tetA*	*Tet*(31) efflux pump, major facilitator superfamily (MFS) transporter
4	AVM72_16315	*tetR*	Trascriptional regulator
5	AVM72_16320	*tnp*	IS*5*/IS*1182* transposase
6	AVM72_16325	*cat2*	Chloramphenicol acetyltransferase
7	AVM72_16330	*tnp*	IS*5*/IS*1182* transposase
8	AVM72_16335		Hypothetical protein
9	AVM72_16340	*aadA1*	Adenylyltransferase, aminoglycoside resistance
10	AVM72_16345	*sat1*	Streptothricin acetyltransferase
11	AVM72_16350	*int*	Integrase, integron class 2-associated
12	AVM72_16355		Hypothetical protein
13	AVM72_16360	*int*	Integrase
14	AVM72_16365		Hypothetical protein
15	AVM72_16370		Restriction endonuclease
16	AVM72_16375	*parB*	Chromosome partitioning protein ParB
17	AVM72_16380	*parA*	ATPase, ParA family protein
18	AVM72_16385	*korA*	Transcriptional regulator KorA
19	AVM72_16390		Hypothetical protein
20	AVM72_16395	*kfrA*	KfrA plasmid-stabilizing protein
21	AVM72_16400		Antirestriction protein
22	AVM72_16405	*repB*	Replication protein
23	AVM72_16410		HigA family addiction module antidote protein
24	AVM72_16415	*traX*	Conjugal transfer protein TraX
25	AVM72_16420		Resolvase
26	AVM72_16425	*traN*	Conjugal transfer protein TraN
27	AVM72_16430		Hypothetical protein
28	AVM72_16435		Hypothetical protein
29	AVM72_16440	*traL*	Conjugal transfer protein TraL
30	AVM72_16445		Hypothetical protein
31	AVM72_16450	*topA*	DNA topoisomerase I
32	AVM72_16455		Hypothetical protein
33	AVM72_16460		Type 4 secretion protein
34	AVM72_16465		Type 4 secretion protein
35	AVM72_16470		Type 4 secretion protein
36	AVM72_16475		Hypothetical protein
37	AVM72_16480		Type 4 secretion protein
38	AVM72_16485		Hypothetical protein
39	AVM72_16490		Type 4 secretory pathway component
40	AVM72_16495		Type 4 secretion protein
41	AVM72_16500		Type 4 secretion protein
42	AVM72_16505	*virB11*	Type 4 secretion system protein VirB11
43	AVM72_16510		Type 4 secretion protein
44	AVM72_16515		Hypothetical protein
45	AVM72_16520	*traD*	Conjugal transfer protein TraD
46	AVM72_16525		Hypothetical protein
47	AVM72_16530		Hypothetical protein
48	AVM72_16535		DNA-binding protein
49	AVM72_16540	*virD2*	Type 4 secretion system protein VirD2
50	AVM72_16545	*mobC*	Mobilization protein MobC
51	AVM72_16550		Hypothetical protein
52	AVM72_16555		Hypothetical protein

Conventionally, the determination of the plasmid incompatibility (Inc) group is based on the amino acid sequence of the replication protein Rep, a key component of plasmid replication systems (Shintani et al., 2015). Comparative analyses of the *rep* sequence of p3PS10 (WP_012850373) showed a perfect match with the corresponding *rep* gene of pEIB202, a plasmid that has been classified as a member of the IncP group (Wang et al., 2009). Since components of the partition system of p3PS10 are also related to the IncP group (Siddique and Figurski, 2002), we postulate that p3PS10 belongs to this same Inc group.

**Figure 2:**
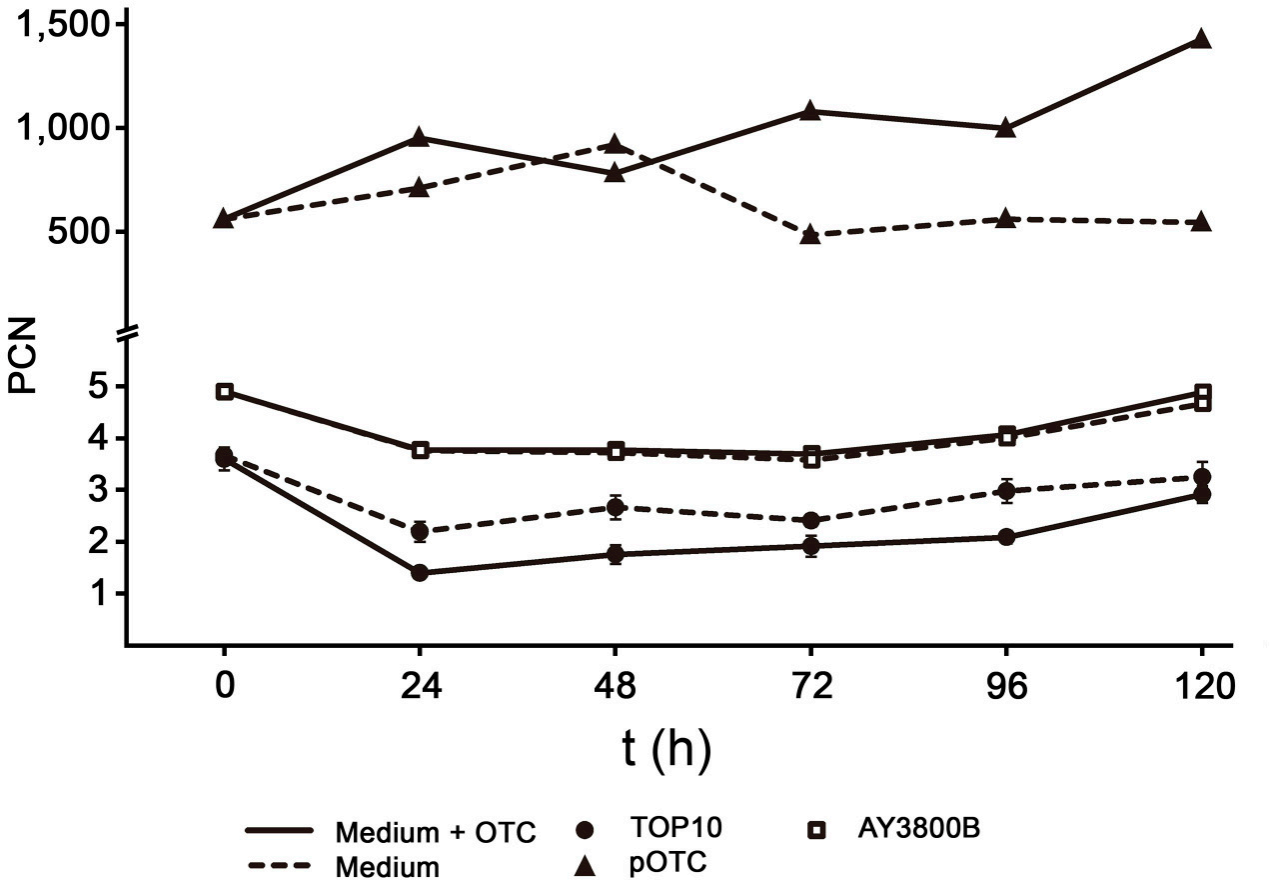
PCN for p3PS10 in AY3800B (unfilled squares) and *E. coli* TOP10 (black circles) host strains cultured in PSB or TSB medium supplemented (continuous line) or not (dashed line) with 50 μg/mL of OTC, respectively. *E. coli* pOTC, which is carrying a pCR2.1 derivative, is shown as control (black triangles).

Concerning the PCN of p3PS10, we predict the value to be low and rest our appraisal on the yield obtained by midipreps (Supplementary Figure 1). Indeed, qPCR-based calculations indicated an average PCN of 3-5 in *P. salmonis* cells (Figure 2). This low PCN was also observed in the heterologous host *E. coli* p3PS10, but not in the control strain *E. coli* pOTC, which carries a high-copy-number pCR2.1 derivative. In this strain, PCN of pOTC exceeded 1,000, as can be expected for this type of plasmid (Invitrogen). Strikingly, PCN of p3PS10 remained constant even after 120 h (~12 generations) of culture in the absence of selective pressure, irrespective of the host. The same observation was made after 60 generations of cultivation (not shown). In contrast, PCN of pOTC started to decline after 48 h in medium without antibiotic.

### The plasmid is able to be transferred into and replicate in homo- and heterologous hosts

Bioinformatic analyses disclosed that p3PS10 encodes a conjugative T4SS gene cluster, which may permit its transmission to different hosts. This possibility was tested in mating assays. These experiments revealed that the donor strain AY3800B is able to transfer the plasmid into homologous hosts. In fact, our results show that the plasmid was mobilized between *P. salmonis* isolates displaying different antibiotic susceptibility patterns (Table 4). Remarkably, p3PS10 was able to replicate within an EM-90-like isolate, representative of a genogroup that has only shown wild-type susceptibility for the major classes of antibiotics utilized for SRS treatment (Saavedra et al., 2016). We calculated the frequencies of plasmid transference and confirmed that such events are rare when donor and recipient derive from different genetic backgrounds, e.g., when LF-89-like AY3800B was paired with EM-90-like PM15972A1. In contrast, transfer frequencies were one or even two orders of magnitude higher for mating pairs with similar genetic backgrounds: in AY3800B-PM63907 and AY3800B-PM32597B1 mating assays, donors and recipients belonged to the LF-89 genogroup (Otterlei et al., 2016). The plasmid was also transferred to the heterologous recipient *E. coli*. In this species, the formation of transconjugants occurred at a frequency similar to that observed in *P. salmonis* mating pairs with the LF-89 background. Antibiotic-resistant colonies were considered as true transconjugants since no spontaneous mutants with reduced susceptibility to either CFC, OTC, SXT, or STR were detected in recipient backgrounds.

**Table 4:** Frequencies of transfer of p3PS10.

**Mating pair**	**Conditions for transconjugants recovery**	**Frequency of transfer^a^**
AY3800B-PM15972A1	PSA, OTC 50 μg/mL, 22°C^b^	1.3 × 10^−7^
AY3800B-PM63907	PSA, oxolinic acid 5 μg/mL+ OTC 50 μg/mL, 18°C	5.3 × 10^−6^
AY3800B-PM32597B1	PSA, oxolinic acid 5 μg/mL+ OTC 50 μg/mL, 18°C	4.2 × 10^−5^
AY3800B-*E. coli* TOP10	TSA, OTC 50 μg/mL, 22°C^c^	3.3 × 10^−6^

a *Average value from three independent experiments. ^b, c^The donor strain AY3800B belongs to the LF-89 genogroup and does not grow at temperatures >19°C*.

### Antibiotic resistance genes carried by the plasmid are functional

Based on our results of sequence analyses and the detection of multiple resistance traits, we decided to characterize the susceptibility of p3PS10-carrying *P. salmonis* AY3800B to OTC, FFC, CFC, STR, and SXT by means of MIC assays (Table 5). The comparison of MIC values for AY3800B with those obtained for the wild-type *P. salmonis* LF-89 reference strain showed that the AY3800B strain was indeed less susceptible to OTC, CFC, and STR, while MIC for SXT were high in both strains. Interestingly, the *E. coli* TOP10 derivative resulting from the transference of p3PS10 exhibited MIC values comparable to those measured for the donor strain AY3800B, which indicates the functionality of resistance markers carried by the plasmid. To confirm this hypothesis, resistance markers for OTC and CFC were cloned into *E. coli* TOP10. For both antibiotics tested, the phenotypic traits of the modified strains were consistent with the bioinformatically predicted function of the corresponding CDS found in p3PS10. In fact, MIC of OTC and CFC were 1,024 and 512 μg/mL in *E. coli* pOTC and *E. coli* pCFC, respectively, which are values similar to those obtained in the *E. coli* p3PS10 strain. The fact that *E. coli* pCFC did not lose susceptibility to FFC, a halogenated CFC derivative, after the transfer of p3PS10, suggests that the reduced susceptibility to CFC is mediated by an acetyltransferase selective for this drug. Due to the intrinsically high *in vitro* MIC of STR for *E. coli* TOP10, we chose to clone the predicted resistance marker for STR into the STR-susceptible *E. coli* DH5α. This way, we were able to show the functionality of this marker, too. With regards to susceptibility to SXT, *P. salmonis* isolates proved to be resistant to this compound irrespective of the presence of p3PS10. Nevertheless, cloning of sulfonamide resistance determinant *sul2* into *E. coli* TOP10 yielded *E. coli* pSUL2, which displayed a significantly decreased susceptibility to SXT.

**Table 5:** MIC for distinct antimicrobials (μg/mL).

**Strain ID**	**OA**	**OTC**	**FFC**	**CFC**	**STR**	**SXT**
LF-89	0.06	0.06	0.25	0.5	8.0	512
AY3800B	0.06	256	1.0	128	>1,024	>1,024
PM15972A1	0.125	0.25	0.5	0.5	16.0	512
PM32597B1	8.0	0.5	2.0	4.0	8.0	512
PM63907	8.0	0.5	1.0	1.0	4.0	>1,024
PM15972A1 p3PS10	0.125	128	2.0	128	>1,024	>1,024
PM32597B1 p3PS10	8.0	128	2.0	128	>1,024	1,024
PM63907 p3PS10	8.0	256	2.0	128	>1,024	>1,024
*E. coli* TOP10	0.25	1.0	4.0	4.0	1,024	1.0
*E. coli* DH5α	2.0	2.0	8.0	8.0	1.0	1.0
*E. coli* ATCC 25922^a^	0.125	1.0	8.0	8.0	2.0	4.0
*E. coli* p3PS10	–	512	4.0	128	1,024	>1,024
*E. coli* pOTC	–	1,024	8.0	4.0	1,024	1.0
*E. coli* pCFC	–	2.0	8.0	512	1,024	1.0
*E. coli* pSTR	–	2.0	8.0	8.0	1,024	1.0
*E. coli* pSUL2	–	2.0	8.0	4.0	1,024	>1,024

a *Control values according to CLSI document VET03/VET04-S2 (2014) for E. coli 25922 incubated at 22°C for 48 h: 0.06–0.25 μg/mL for oxolinic acid (OA), 0.5–2.0 μg/mL for oxytetracycline (OTC), 4.0–16.0 μg/mL for florfenicol (FFC), 0.25–4.8 μg/mL for sulfamethoxazole/trimethoprim 19:1 (SXT). MIC for streptomycin (STR) and chloramphenicol (CFC) are not informed*.

### p3PS10 might have been acquired in a horizontal gene transfer event

The sequencing of *P. salmonis* AY3864B and AY6297B showed that both isolates harbor an identical p3PS10 plasmid that has been given different names during automatic annotation (p3PS11 and p4PS8, respectively), whereas *P. salmonis* AY6532B carries p4PS9, a smaller plasmid encompassing 20.4 kb and lacking transfer genes, sulfonamide and TET resistance markers (Supplementary Figure 2, Supplementary Table 3). BLASTN analysis disclosed that 35 kb of the backbone of p3PS10 matched with an identity of more than 99% to the sequence of the 43.7-kb plasmid pEIB202 found in the Gram-negative bacterium *Edwarsiella tarda* strain EIB202, the causative agent of the fish disease edwardsiellosis (Wang et al., 2009). Both p3PS10 and pEIB202 show similar patterns of genetic organization and comprise sequences encoding multidrug resistance markers, genes required for replication, and a set of T4SS genes. Nevertheless, the 11-kb locus of p3PS10 that harbors resistance genes clearly distinguishes this plasmid from pEIB202. The sequence of this locus showed to be partially homologous to a segment of 3.5 kb found in the 48-kb plasmid pRAS2 (accession number AJ250203). pRAS2 was isolated from *Aeromonas salmonicida* subsp. *salmonicida* 1682/92, and constitutes an episome conferring STR, sulfonamide, and TET resistances (L'Abee-Lund and Sorum, 2000).

**Figure 3:**
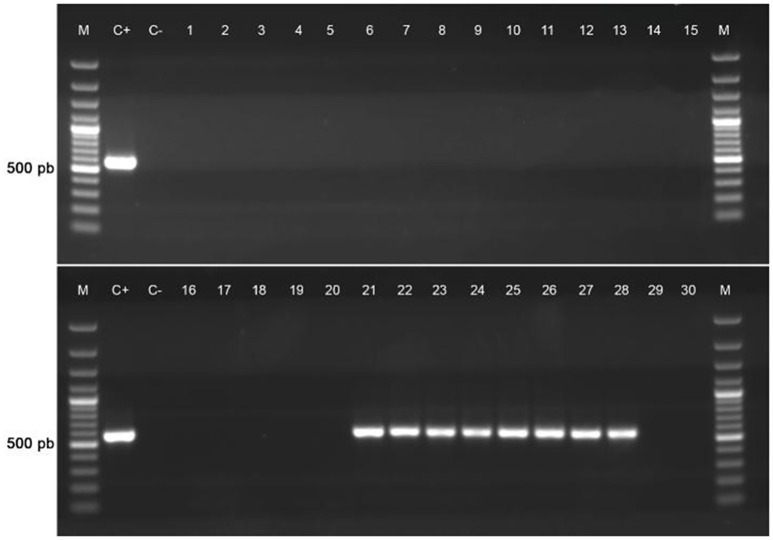
Screening for the *tet*(31) element in a group of representative *P. salmonis* isolates. Epidemiological data of isolates can be reviewed in Supplementary Table 4.

To study the similarity between p3PS10 and known plasmids at the level of amino acid sequences, BLASTX searches were carried out using the sequence of the 11-kb locus of p3PS10. We could identify several components conserved across different bacterial species, e.g., those genes encoding a class 2 integron-associated integrase, aminoglycoside resistance proteins, and a pair of IS*1182* transposases flanking the *cat2* gene (Figure 1B). The class 2 integron sequence conferred resistance to STR and was almost identical to the one detected in the cholera agent *Vibrio cholerae* VC97 (accession number GU570569; da Fonseca et al., 2011). Furthermore, the transposon-like structure in p3PS10 proved to be identical to a locus found in a plasmid carried by a CFC-resistant *Photobacterium damselae* subsp. *piscicida* strain (accession number AB453229), causative agent of pseudotuberculosis in fish (del Castillo et al., 2013). The 2-kb sequence encoding the *tet*-resistance determinant carried by p3PS10 was found in several aquatic pathogens, such as in *A. salmonicida* and *Vibrio splendidus*. Somewhat surprisingly, this sequence was also identified in the genome of *Gallibacterium anatis* strain 12656-12, a bacterium causing septicemia in chicken (Kudirkiene et al., 2013). The *tet*-resistance element was highly prevalent in a collection of *G. anatis* field isolates resistant to tetracycline, which have been gathered in Mexico and Denmark (Bojesen et al., 2011). We investigated the prevalence of the *tet* element in a group of representative isolates from our *P. salmonis* collection (Figure 3). As expected, those isolates carrying *tet*(31) were associated with OTC treatment failures observed in the field (Supplementary Table 4). These isolates were recovered from SRS outbreaks that took place in the south of Región de Aysén, in the period 2013-2015 (Supplementary Figure 3). This restricted dissemination pattern contrasts with the wide distribution reported for non-wild-type isolates for quinolones and FFC (Henríquez et al., 2015).

## Discussion

The use of antibiotics in Chilean aquaculture is largely linked to the trend in productivity observed in the salmon industry. In the period 2014–2015, more than 500 metric tons/year were used to combat SRS outbreaks, with FFC and OTC being the active principles most frequently applied in field therapies (SERNAPESCA, 2015, 2016). Owing to a coordinated effort to reduce the use of antimicrobial drugs in the Chilean salmon industry, this amount dropped by roughly 30% the following year, reaching 382 tons in 2016 (SERNAPESCA, 2017). However, even in this scenario, the probability of the emergence and spread of resistances within the bacterial population surrounding salmon farms is still high. Additionally, in recent years, a continuous trend toward the preferential consumption of FFC was recorded and the relative share of this drug augmented to 81% and 90% in 2015 and 2016, respectively (SERNAPESCA, 2015, 2016). This situation predicts a reduction in the efficacy of therapies in forthcoming years due to the selection of strains with reduced susceptibilities to FFC. As it can be assumed from our results, the transference of p3PS10 to *P. salmonis* strains with a reduced susceptibility to FFC could trigger the worst-case scenario for the veterinary management of piscirickettsiosis outbreaks: so far, *P. salmonis* with a reduced susceptibility to FFC can be controlled applying OTC, but that would no longer be the case. In fact, *P. salmonis* isolates with non-wild-type traits for quinolone and FFC have already been reported (Henríquez et al., 2015; Saavedra et al., 2016). Therefore, the transmissibility and the replicative capacity of p3PS10 in homo- and heterologous hosts demonstrated in this study do not only point out the possibility of the emergence of *P. salmonis* strains displaying additional resistances, but also warn against the formation of other multidrug-resistant pathogens. An interesting point was the difference in p3PS10 transfer frequencies between *P. salmonis* isolates from identical and distinct genetic backgrounds. In our experiments, the transfer from LF-89-like to EM-90-like isolates occurred at a lower frequency, possibly because the latter group is refractory to the uptake of DNA. The fact that our actual collection of over 600 field isolates, with more than 50% of them being EM-90-like specimens, is lacking non-wild types belonging to this genogroup seems to support this theory. More research is needed on additional EM-90-like isolates to prove – or refute –this hypothesis.

This study further adds to our understanding of clinically important traits of *P. salmonis*. It is also the first description of antimicrobial resistances encoded by and mobilized in a plasmid of this pathogen. The medium-sized plasmid p3PS10 apparently replicates in a broad-host-range manner, while being preserved as a low-copy-number element, which reduces the fitness burden on its bacterial host. Intriguingly, the plasmid is maintained even in the absence of selective pressure. This finding strongly suggests that it carries a functional toxin-antitoxin (TA) addiction module, a genetic element whose expression facilitates the postsegregational killing of plasmid-less daughter cells in order to conserve the episome through generations (Unterholzner et al., 2013). TA modules were reported to be transcriptionally active in the LF-89 strain (Gómez et al., 2011), which makes it seem likely that similar preserving strategies are pursued by other plasmids found in *P. salmonis*. The significance of this mechanism lies in the persistence of resistance traits in the bacterial community even when the selective pressure has been removed from the environment.

Sequencing data suggest that p3PS10 has been horizontally acquired. The GC content of the plasmid discloses a genomic signature distant from its host *P. salmonis* (55 and 39%, respectively), while its mobilization was confirmed by mating assays. Despite the overwhelming sequence identity between the backbones of plasmids found in fish pathogens *P. salmonis* and *E. tarda*, genetic divergence was evident regarding the locus encoding multidrug resistance. This finding may be explained by the composite structure of the 11-kb locus, which probably consists of recombination-prone, independently mobilizable DNA fragments, a feature that can be extended to the *tet*-resistance cassette found in p3PS10. A homologous *tet*(31) element was found in pRAS2, an episome of *A. salmonicida*, a Gram-negative bacterium that causes furunculosis in salmonids. pRAS2 and its relative pRAS1 have been related to resistances to TET as well as trimethoprim and sulfonamides, and such resistances are exhibited by a series of *A. salmonicida* isolates recovered in Norwegian fish farms in the late 1980s (L'Abee-Lund and Sorum, 2000; Sørum et al., 2003). It is equally conceivable that p3PS10 has been acquired from environmental bacteria. In fact, Miranda et al. (2003) described environmental microorganisms able to tolerate OTC at concentrations ranging between 128 and 2,048 μg/mL. These MIC values are comparable to those described in the present study. However, the sequences of these *tet* genes do not match with that of *tet*(31), which was found in p3PS10 and its relatives. The fact that the *P. salmonis tet*-resistance determinant shows a high degree of sequence identity with aquatic and terrestrial pathogens such as *G. anatis* reflects the ubiquity of such resistance elements, providing an example of the flux of ARG between distinct environments.

According to our results, the mechanism underlying OTC resistance examined by us is different from that recently described by Contreras-Lynch et al. (2017). In the cited work, *P. salmonis* with reduced susceptibility to OTC were defined as those isolates tolerating OTC concentrations of >0.5 μg/mL, and MIC values of only 1.0–4.0 μg/mL were obtained for representatives of this category. By contrast, the group of isolates studied here exhibited significantly higher MIC to OTC and these MIC correlated with resistance from a clinical point of view. One possible explanation for these differences is that alternative mechanisms operate in those non-wild types reported by Contreras-Lynch and colleagues, such as the TET efflux pumps identified in a previous study (Cartes et al., 2016).

The phenotype linked to the resistance markers for CFC and SXT, as predicted after antibiotic resistance ontology analyses performed with CARD, deserves special attention. According to the results of these analyses, both the genomes of *P. salmonis* LF-89 and AY3800B contain five copies of chromosomal genes encoding CFC resistance-related efflux pumps, and AY3800B additionally carries *cat2*. However, and regardless of the redundancy, it seems that only the specific acetyltransferase encoded by *cat2* in p3PS10 confers resistance to CFC. This conclusion is based on MIC values obtained for CFC-susceptible *P. salmonis* and resistant *E. coli* pCFC strains and stresses the need for a careful interpretation of the bioinformatic prediction. Concerning the resistance to sulfonamides, the prediction did not result as straightforward. The putative resistance marker *sul2* was identified in the SXT-resistant AY3800B strain, but a similar phenotype was also displayed by the *sul2*-lacking LF-89 background. These findings reflect a lower sensitivity of CARD analysis for this trait. Thus, the genetic background of reduced susceptibility to SXT, as exhibited by all *P. salmonis* strains used in this study, requires further examination.

In summary, our results shed light on the plasmid biology and the genetic basis of antibiotic resistance in *P. salmonis*. This pathogen can be considered a dissemination vector for ARG in the seawater ecosystem, a role played by this insidious bacterium that has been underestimated so far. However, the low transfer frequency detected for p3PS10 makes its dissemination directly to human pathogens a rather unlikely event. The plasmid described in this work may serve as a valuable genetic tool for upcoming basic research on piscirickettsiosis, but also for future biotechnological applications focused on the control of this important fish pathogen.

## Author contributions

JS carried out PCR assays, molecular cloning, mating assays, and helped with the preparation of the figures. MG phenotypically characterized bacterial strains and performed PCN experiments. JV-G accomplished the restriction analyses and assisted with mating assays. HB designed qPCR primers and probes, assembled and annotated the sequencing data, and carried out CARD analyses. PB aided to obtain financial support and to draft the manuscript. MM conceived the study, performed sequence analyses and wrote the manuscript. All authors read and approved the final version of the text.

### Conflict of interest statement

The authors declare that the research was conducted in the absence of any commercial or financial relationships that could be construed as a potential conflict of interest.

## Footnotes

^1^http://arpcard.mcmaster.ca.

^2^http://frodo.wi.mit.edu/.
