# Supplementary material for: Isolation, Functional Characterization and Transmissibility of p3PS10, a Multidrug Resistance Plasmid of the Fish Pathogen Piscirickettsia salmonis
Source: Front Microbiol. 2018 May 8;9:923. doi: 10.3389/fmicb.2018.00923 (PMC5952111; doi:10.3389/fmicb.2018.00923)
Supplement: Supplementary file 1 [file Table_1.pdf]

frontiers

Supplementary Material

Isolation, functional characterization and transmissibility of p3PS10, a multidrug resistance plasmid of the fish pathogen *Piscirickettsia salmonis*

José Saavedra, Maritza Grandón, Juan Villalobos-González, Harry Bohle, Patricio Bustos, Marcos Mancilla*

*** Correspondence:** Corresponding Author: mmancilla@adldiagnostic.cl

Supplementary Table 1. Statistics of PacBio and Illumina sequencing for additional OTC-resistant *P. salmonis* isolates.

Strain ID	AY3864B			AY6297B			AY6532B		
Replicons	Accession n°	ID	Size (bp)	Accession n°	ID	Size (bp)	Accession n°	ID	Size (bp)
Chromosome	CP013811	Chr	3187888	CP013791	Chr	3187881	CP013796	Chr	3186791
Plasmid 1	CP013812	p1PS11	60086	CP013792	p1PS8	188268	CP013797	p1PS9	188297
Plasmid 2	CP013813	p2PS11	33556	CP013793	p2PS8	60081	CP013798	p2PS9	60078
Plasmid 3	CP013814	p3PS11	45767	CP013794	p3PS8	33557	CP013799	p3PS9	33518
Plasmid 4	CP013815	p4PS11	188300	CP013795	p4PS8	45766	CP013800	p4PS9	20368
Total CDS	3273			3272			3230		
N° PacBio mapped reads	56405			80995			60443		
Average length coverage	140.4x			223.95x			185.95x		
N_{50}	20948			18885			23273		
N° Illumina pair-end reads	61823378			-			-		
Average length coverage	1688.05x			-			-		

Supplementary Table 2. CARD analyses of *P. salmonis* LF-89 (VR-1361) and AY3800B genomes (Excel file).

Supplementary Table 3. Features of *P. salmonis* isolates carrying p3PS10-related plasmids according to genome sequences retrieved from GenBank. All isolates were recovered from Atlantic salmon, *Salmo salar*.

Strain ID	Plasmid ID	Accession number	Size (kb)	Coverage (%)	% identity to p3PS10	TET-resistance marker *tet*(31)	MIC of OTC (µg/mL)	MIC of CFC (µg/mL)
AY3864B	p3PS11	CP013814	45.7	100	100	+	128	128
AY6297B	p4PS8	CP013795	45.7	100	100	+	256	128
AY6532B*	p4PS9	CP013800	20.4	44	100	-	32	32

*AY6532B arised as a mixed culture in which the *tet*-resistance element was initially detected, but further incubation under non-selective conditions allowed to isolate OTC-susceptible clones.

Supplementary Table 4. Epidemiological data of *P. salmonis* isolates screened for the presence of the OTC-resistance marker *tet*(31) as shown in Figure 3. Isolates with a reduced susceptibility to OTC are highlighted in red.

Lane	ID	Lineage	Year of isolation	Geographic origin[a]	Host	OTC MIC (µg/mL)	OTC treatment outcome[b]
1	PM15972A1	EM-90	2010	28a	*S. salar*	0.25	
2	PM-21657	EM-90	2011	18c	*S. salar*	0.13	
3	PM-23019	EM-90	2011	17b	*S. salar*	0.13	
4	PM-24231	EM-90	2011	18c	*S. salar*	0.25	
5	PM-26862	EM-90	2011	2	*S. salar*	0.13	
6	PM-37984	EM-90	2013	17a	*S. salar*	0.25	
7	AY-6492	LF-89	2015	21d	*S. salar*	0.50	
8	PM-19142	LF-89	2010	3b	*O. mykiss*	0.50	
9	PM-23150	LF-89	2011	17b	*O. mykiss*	0.50	
10	PM-29917	LF-89	2012	1	*O. mykiss*	0.25	
11	PM-33063	LF-89	2012	17b	*O. mykiss*	0.50	
12	PM-34720	LF-89	2012	16	*O. mykiss*	0.50	
13	PM-39637	LF-89	2013	16	*S. salar*	0.50	
14	PM-41864	LF-89	2013	11	*O. mykiss*	0.50	
15	PM-43732	LF-89	2013	1	*O. mykiss*	0.50	
16	PM-31429	LF-89	2012	17b	*O. mykiss*	1.00	
17	PM-49811	LF-89	2014	17b	*S. salar*	0.50	
18	PM-58386	LF-89	2015	16	*S. salar*	1.00	
19	PM-22180	LF-89	2011	32	*O. mykiss*	0.25	
20	PM-43955	LF-89	2013	2	*S. salar*	0.50	
21	AY3800B	LF-89	2013	23b	*S. salar*	256.0	Failure (oral)
22	AY6297B	LF-89	2015	27	*S. salar*	256.0	Not available
23	PM-59281	LF-89	2015	27	*S. salar*	256.0	Not available
24	AY-4709	LF-89	2014	23c	*S. salar*	128.0	Failure (injectable)
25	PM-56204	LF-89	2015	23a	*S. salar*	256.0	Failure (injectable)
26	AY-3635	LF-89	2013	23b	*S. salar*	128.0	Failure (oral)
27	AY-5261	LF-89	2014	23a	*S. salar*	128.0	Failure (oral)
28	PM-56155	LF-89	2015	24	*S. salar*	256.0	Failure (oral)
29	PM32597B1	LF-89	2012	17b	*O. kisutch*	0.50	
30	LF-89	LF-89	1989	3a	*O. kisutch*	0.06	

[a]According to the official classification informed by the regulatory authority, which group several marine farms for each *barrio* (http://www.subpesca.cl/portal/619/w3-article-81329.html). [b]OTC prescriptions: injectable, a single dose of 1.0 mL of 20% suspension to be injected intraperitoneally; oral, 100 mg drug/kg biomass for 10-14 days. Failure means that treatments neither halted nor stabilized mortality caused by SRS.
