# Supplementary material for: Isolation, Functional Characterization and Transmissibility of p3PS10, a Multidrug Resistance Plasmid of the Fish Pathogen Piscirickettsia salmonis
Source: Front Microbiol. 2018 May 8;9:923. doi: 10.3389/fmicb.2018.00923 (PMC5952111; doi:10.3389/fmicb.2018.00923)
Supplement: Supplementary file 3 [file Image_1.PDF]

Supplementary Material

Isolation, functional characterization and transmissibility of p3PS10, a multidrug resistance plasmid of the fish pathogen *Piscirickettsia salmonis*

José Saavedra, Maritza Grandón, Juan Villalobos-González, Harry Bohle, Patricio Bustos, Marcos Mancilla*

*** Correspondence:** Corresponding author: mmancilla@adldiagnostic.cl

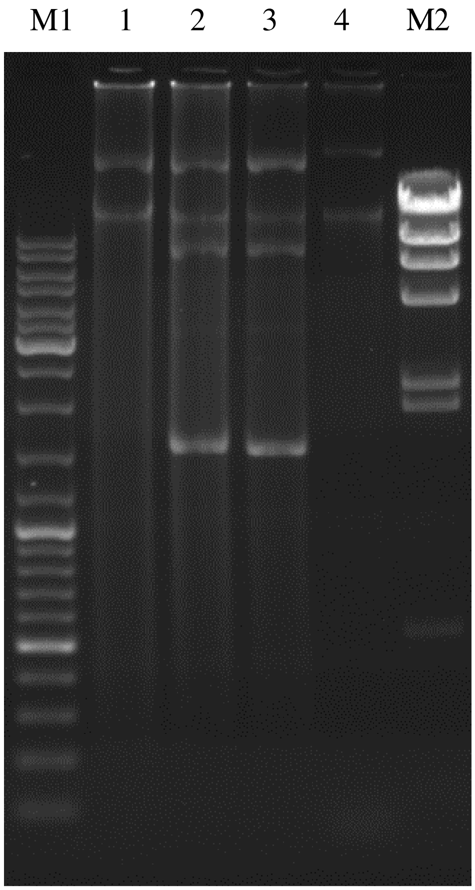

Supplementary Figure 1: Plasmid profile of recipient strains used in mating assays. 7 µL of each midiprep representing 90 ng were resolved. Lanes 1-3, *P. salmonis* PM15972A1, PM32597B1, PM63907; lane 4, *E. coli* p3PS10. M1, 10 kb DNA ladder; M2, λ phage/*Hind*III ladder (500 ng).

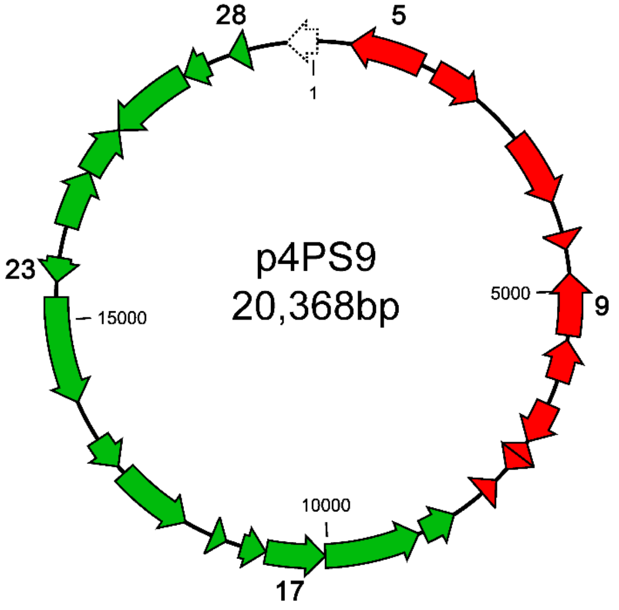

Supplementary Figure 2: Genetic organization of p4PS9 present in the AY6532B. Numbers refer to CDS listed in Table 3.

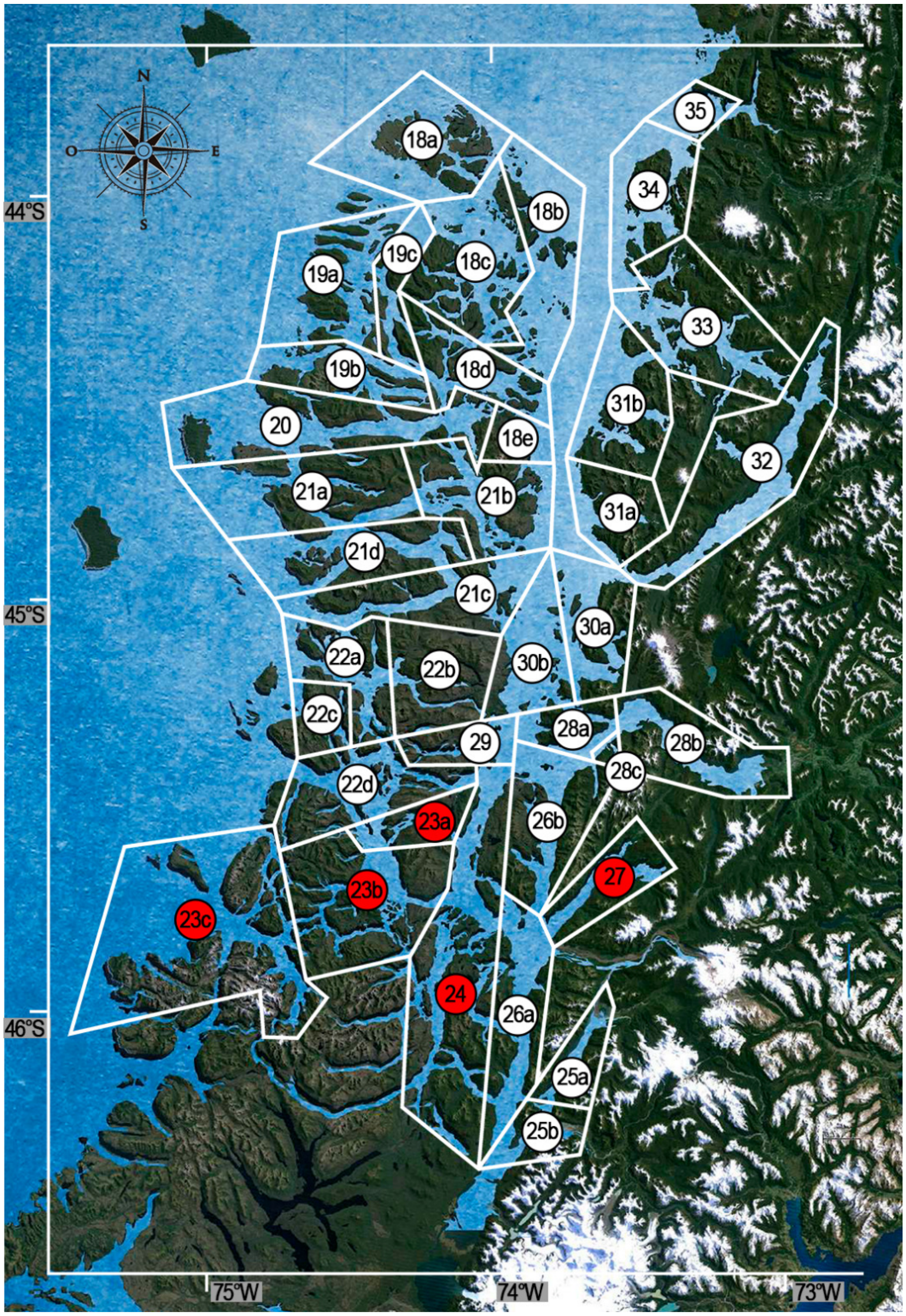

Supplementary Figure 3: Map of Región de Aysén showing *barrios* or maritime concessions. Areas where isolates with a reduced susceptibility to OTC were recovered are marked with a red point. Epidemiological data related to isolates can be seen in Supplementary Table 4.
